# Impact of Hepatitis C on Survival of HIV-Infected Individuals in Shiraz; South of Iran

**DOI:** 10.5812/hepatmon.839

**Published:** 2012-02-29

**Authors:** Abbas Rezaianzadeh, Jafar Hasanzadeh, Abbas Alipour, Mohamed Ali Davarpanah, Abdorreza Rajaeifard, Seyed Hamid Reza Tabatabaee

**Affiliations:** 1 Research Center for Health Sciences, Epidemiology Department, School of Health and Nutrition, Shiraz University of Medical Sciences, Shiraz, IR Iran

**Keywords:** HIV, Coinfection, Survival Rate

## Abstract

**Background:**

HIV and HCV infections are basic issues of many health systems. Since HIV and HCV are transmitted similarly, it is common to become infected by them simultaneously. No consensus exists on the effect of HCV infection on the survival of HIV-infected patients.

**Objectives:**

This study aimed to investigate the issue in a relatively large cohort of patients who had a high prevalence of this coinfection in Shiraz (South of Iran).

**Patients and Methods:**

In this historical cohort study, we evaluated the survival time of 1338 HIV-infected individuals who had been referred to a behavioral consultation center in Shiraz over 10 years (from April 2001 to July 2011). Kaplan-Meier method and log-rank test were used to investigate patient survival and compare their survival curves, respectively. Moreover, Cox proportional hazards model was used to examine the effect of HCV infection on patient survival after control for age, sex, having the injection drug use (IDU) risk factor, CD4 count at baseline, more than a 30% decline in CD4 cell count, and highly active antiretroviral therapy (HAART).

**Results:**

In our cohort, 1044 patients (78.03%) were infected by HCV. The median follow-up was 43.48 months (95% CI = 61.18-26.63). The median survival time in HCV-infected and uninfected patients was 163.8 and 194.8 months, respectively (P = 0.039). After controlling for other covariates, HCV infection increased the mortality rate 2.13 times more in HCV-infected patients than HCV -uninfected patients (CI: 95%; 1.1-4.52).

**Conclusions:**

HCV infection increases AIDS-related deaths. To control HCV infection and transmission and eliminate HCV, timely diagnosis and treatment and serious harm reduction programs must be implemented.

## 1. Background

HIV and HCV infections are basic issues that are faced by health systems in many societies. Because HIV and HCV are transmitted in similar ways, it is common to become coinfected by them [1][2][3]. It is estimated that 34-38.6 million people around the world are infected by HIV compared with 170-200 million by HCV and 4-5 million people who are coinfected with HIV and HCV [4][5]. It is also estimated that 68,000-110,000 people [6] (average = 86,000, less than 0.2%) in Iran are infected by HIV [7], and less than 1% is infected by HCV [8]. The prevalence of HIV and HCV coinfection depends on facilitating risk factors, such as IDU, and varies between countries-even between various areas within a country [9][10][11][12]. Surveys show that the prevalence of this coinfection in HIV-infected individuals who have a background of IDU is 92%, 91%, and over 90% in the US [13], Russia [14], and Asia [12], respectively. Although many studies have shown that HIV infection leads to a decrease in HCV clearance and increases in viral load and complications from the infection [15][16][17], no consensus exists regarding the effect of HCV infection on the natural process of HIV infection. Some studies have revealed that this coinfection affects HIV progression and its related mortality [18][19]. Other studies, however, have not shown the same results-especially after the wide use of HAART [20][21]. In other words, considering HAART and controlling its effects, no consensus exists regarding the effect of HIV infection on general survival or HIV-related survival in HIV patients [22]. In addition, few studies have been conducted on the survival of HIV patients and its factors in Iran.

## 2. Objectives

This study aimed to investigate the effect of HCV infection-after controlling for the effects of HAART-on survival in a relatively large cohort of HIV patients who have a high prevalence of HCV coinfection in Shiraz (South of Iran).

## 3. Patients and Methods

The registration of HIV patients who participated in this cohort study began in April 2001. These patients were diagnosed as definite HIV infections (by serial ELISA and western blot) among thousands of individuals referred to this center due to risky behaviors. In the center, HIV-infected patients receive free services, such as regular laboratory and radiological exams, medications prescribed by a physician, and personal and familial consultations. The interviewers were well trained and familiar with the patients under study. Also, the quality control of the data was done by a researcher. Through July 15, 2011, 1683 HIV patients took part in this study. The participation criteria included being visited twice by an infectious disease specialist in the past 18 months, determination of their survival status in the past 6 months, age over 17 years, and to be in the cohort study at least for 12 months. Accordingly, 1338 patients met the criteria. HAART treatment was performed for some patients in this center in 2003. Since 2004, however, it has been done routinely for eligible patients, based on WHO instructions and their adjusted forms, which were prepared and supplied by the Iranian Ministry of Health and Medical Education (this treatment does not differ between HIV infected and HIV/HCV coinfected subjects). The causes of participants' deaths were gathered from their profiles and confirmed by the general practitioner of the center, who was familiar with the patients. In cases for which the status of death was not known, the center's social work department called the intended patients or their families and confirmed the patient's survival status. If a patient was dead, the exact cause of death was determined and recorded through hospital profiles or the death certificate. Then, the causes of definite HIV- and AIDS-related deaths were identified and examined as the event of the study [23]. A venous sample was taken by a professional phlebotomist from the arms of all subjects who participated in the blood test. By ELIZA, the samples were screened for anti-HIV (Delaware Biotech Inc., USA), and the diagnosis of HIV was confirmed by western blot (Diagnostic, Germany). A third-generation ELISA (Dia. Pro, Italy) was used to determine anti-HCV. Positive results from both the western blot (for HIV) and third-generation ELISA (for HCV) were considered coinfection with HIV and HCV. CD4+ cell counts were measured by flow cytometry (Partec, Germany). The project was approved by the vice chancellor for research affairs, Shiraz University of Medical Sciences.

Two periods were defined for the patients: before HAART (before 2004) for patients who had been registered before 2004 and during HAART for patients who had been registered after 2004. The patients were categorized into 2 groups: those who were infected with HCV and those who were not. The characteristics of the study participants were compared by chi-square test and t-test regarding the qualitative and quantitative variables, respectively. The mortality rate from AIDS-related complications was calculated by dividing the number of deaths by time-persons of follow-up in the subgroups; 95% confidence intervals were also calculated. Survival curves of the 2 groups were drawn and compared by Kaplan-Meier method and log-rank test. Moreover, Cox proportional hazards model was used to examine the effect of HCV infection on patient survival, controlling for age, sex, IDU, CD4 count at baseline, a greater than 30% decline in CD4 cell count during the study, and HAART treatment. The assumption of analysis in the Cox model was evaluated using parallel survival curves. In the survival analyses, the time of patient registration and entrance into the cohort was considered the starting point (T0), and AIDS related death was considered the event of the study. Statistical analyses were performed using SPSS (version 16) and Stata (version 10).

## 4. Results

In this study, 1338 HIV patients-204 (15.2%) of whom were women-were investigated. The median age was 36 [32][33][34][35][36][37][38][39][40][41][42] years; 1044 (78.03%) of these patients were infected by HCV. The median follow-up was 43.48 (61.18-26.63) months. Other characteristics of the population, based on HCV status, are shown in Table 1.

**Table 1:** Demographic and Baseline Characteristics of the Study Cohort Divided Into the Groups Infected With Hepatitis C Virus and Not Infected With Hepatitis Virus C

	**Patients, (n = 1338)**	**HCV Group, (n = 1044)**	**Non-HCV Group, (n = 294)**	***P* value**
Females, No. (%)	204 (15.2)	36 (3.4)	168 (57.1)	< 0.0001
Age, median (range)	36 (32- 42)	37 (32- 43)	35 (31- 42)	0.002
IDU risk factor, No. (%)	987 (73.8)	918 (87.9)	69 (23.5)	< 0.0001
Fallow up time in month, median (range)	43.48 (26.23 – 61.58)	45.33 (27.74- 62.5)	37.3 (20.6- 54.8)	0.006
CD4 cell count at base line ≤ 200 cells/μL, No. (%)	306 (22.9)	224 (21.5)	82 (27.9)	0.78
> 30% decline in CD4 cell count at fallow up time, No. (%)	145 (10.8)	106 (10.2)	39 (13.3)	0.89
HAART therapy, No. (%)				0.75
No (before 2005)	134 (10)	106 (10.2)	28 (9.5)	
Yes (after 2005)	1204 (90)	938 (89.8)	266 (90.5)	

Most of the HCV infected individuals were males and older and had risk factors of more IDU and longer follow-ups in the study. The rate of AIDS-related deaths decreased to 74.8% after 2004 and from 5.59 (95%CI: 3.93-7.96) to 1.41 (95% CI: 1.1-1.82) deaths per 100 person-years (P < 0.0001). The rate of AIDS-related deaths increases 23.23% in people with HIV/HCV coinfection compared with patients with HIV. The mortality rate in patients with HIV/HCV coinfection has been 1.98 (95% CI: 1.59 - 2.48) deaths in 100 person-year, while it has been reported as 1.52 (95% CI: 0.92 - 2.52) deaths in 100 person-year in only HIV infected patients. (P = 0.03). These rates in HIV/HCV-coinfected persons in the pre-HAART and post-HAART periods were 5.51 (95% CI: 3.62-8.37) and 1.58 (95% CI: 1.21-2.06) per 100 person years, respectively (P < 0.0001). Also, the mortality rate was compared between the patients with baseline CD4 counts below and above 200, which showed an 82.33% increase in patients with baseline CD4 counts below 200. The mortality rate of the first group (patients with baseline CD4 counts below 200) was 4.3 (95% CI: 3.25-5.69) per 100 person-years versus 0.76 (95% CI: 0.47-1.25) per 100 person-years in the second group (patients with baseline CD4 above 200) (P < 0.0001). The median survival time in the HCV-infected and uninfected patients was 163.8 and 194.8 months, respectively (P = 0.039). In the multivariate analysis (Table 2), it was revealed that after controlling for covariates, HCV infection increased the number of deaths in HCV-infected patients 2.13 times (95% CI: 1.1 - 4.52) more than HCV-uninfected patients. Covariates of HAART (aHR = 0.46; 95% CI: 0.24-0.86), CD4 count at baseline (aHR = 5.38; 95% CI: 3.04-9.51), and more than a 30% decline in CD4 count (aHR = 2.93; 95% CI: 1.65-5.2) correlated with survival length in the follow-up (P < 0.05). In order to estimate rates of survival by HCV status according to IDU and sex, 2 Cox analyses were performed with the (HCV × IDU and HCV × sex) interaction terms in the models. The lack of statistical significance between these interactions (P = 0.987 and P = 0.858, respectively) showed that the effect of HCV on HIV survival was not modified by IDU or gender and did not require a separate analysis.

**Table 2:** Multivariate Analysis of the Time to HIV/AIDS Related Death

	**Value or Characteristic Compared**	**Crude Hazards Ratio**	**Adjusted Hazards Ratio**	***P* value**
Age	1 year increasing	0.997 (0.975-1.02)	0.99 (0.96- 1.02)	0.647
Sex	Female vs. male	0.57 (0.28- 1.17)	0.54 (0.2- 1.44)	0.216
IDU	Non IDU vs. IDU	0.996 (0.63- 1.57)	0.84 (0.46- 1.54)	0.57
CD4 cell count at base line	< 200 vs. ≥ 200 cells/μL	4.3 (2.5- 7.2)	5.38 (3.04- 9.51)	< 0.0001
Decline in CD4 cell count during fallow up	Yes vs. No	1.64 (0.95- 2.8)	2.93 (1.65- 5.2)	< 0.0001
HCV	Positive vs. Negative	1.9 (1.1- 3.5)	2.13 (1.1- 4.52)	0.048
HAART	Yes vs. No	0.36 (0.21- 0.59)	0.46 (0.24- 0.86)	0.015

## 5. Discussion

In our large cohort study, the prevalence of HIV/HVC coinfection was 78%. This coinfection was related to IDU background. Intravenous drug use has been the predominant mode of transmission of HCV in the world [24][25], and approximately 75% HIV-positive people in Iran are IDUs [26]. The results of the present study are consistent with previous national [27][28] as well as international [29][30] studies and emphasize IDU as one of the major routes of HVC transmission. Our study revealed that HAART treatment leads to a 54% decrease in AIDS-related deaths. Other studies have also shown that HAART treatment results in a decrease in mortality rate in HIV patients 3 to 10 times more than untreated HIV patients with HAART and changes HIV/AIDS into a chronic disease [31]. The international experience has shown that before commencement of HAART treatment, almost all HIV patients died due to AIDS-related diseases; today, however, almost half of these patients in North America and Europe die from reasons that are not related to AIDS [32]. HAART treatment not only decreases necrotic inflammation and progressive liver diseases [33][34] but also reduces the replication of viruses in the liver [34] and increases the CD4 count [35]. On the other hand, HAART treatment is advised to be performed before beginning HCV treatment, since it may cause fibrosis through cumulative hepatotoxicity in HIV/HCV-coinfected patients.

Regardless, the results of the present study were consistent with the results of other studies, and emphasize the important role of HAART treatment in the survival of HIV patients [36]. As expected, CD4 count at baseline correlated with AIDS-related death. CD4 T cells are the primary target of HIV and an important marker in evaluating the immune system's health. Decline of CD4 cells through increased infections by opportunistic diseases results in an rise in HIV-related deaths [32]. In addition to CD4 count at baseline and last CD4 count before HAART treatment, the duration of a patient's life with a high CD4 count influences his prognosis; a 30% decline in CD4 count during the follow-up shows that patients are more at risk and consequently affects their survival [31][37]. These results are consistent with the results of other researchers and emphasize the importance of the follow-up of patients' CD4 count during HAART [38]. The results of the present study show that HCV infection in HIV-infected patients increases AIDS-related deaths by 2.13 times, consistent with most of the results of other studies [18][39][40]. On the other hand, many studies have not found a relationship between this coinfection and overall death rates before HAART treatment or between this coinfection and AIDS-related deaths after HAART treatment, as shown in a meta-analysis of 30 studies and over 100,000 patients [22]. These variations might be due to the type of HAART treatment, adherence of patients to HAART treatment, different endpoints, various sample sizes, different follow-up times, and ignoring some altering factors. Fortunately, the type of treatment and the definition of AIDS-related death are clear in our population; ie, per the Shiraz Center of Behavioral Consult. The sample size and the follow-up time are also suitable. Although no independent studies have been conducted on the status of patient adherence in this center, primary surveys as well as the experiences of doctors report the 25% to 30% treatment failure, which is in line with other national studies [41]. The treatment failure might be affected by the high proportion of IDU and, consequently, the high proportion of HIV/HCV coinfection, which strongly affects patient adherence to HAART treatment. In spite of the fact that the mechanisms through which HCV infection worsens the condition of HIV patients and increases its complications and AIDS-related deaths are not clear, it likely activates immune cells with the CD4 apoptosis marker and causes a severe defect in the immune system [42], decreasing the power of recovery in CD4 T cells after HAART treatment [43]; increasing the production of cryoglobulin by activation of B cells [44], decreasing the production of CRP [45]; and activating the replication of HCV in lymphoid [46] and lymphoblastic [47] tissues as well as in environmental CD4 T cells in HIV patients [48]. The present study revealed that HAART treatment is highly effective in HIV patients in Iran. In addition, HCV coinfection, as an independent factor, can threaten these patients. These findings should prompt us to prevent HCV infection and eliminate this virus in HIV patients. Conducting programs for screening blood of HCV and HCV RNA antibodies is quite beneficial. Fortunately, this program is being seriously conducted in Iran and, particularly, Fars [49], as well as some developed and developing countries. Harm reduction programs in at-risk populations - particularly HIV patients - is also of great importance. Fortunately, these programs are also being conducted in a high quantity and quality in Iran; in 2007, the Iranian health system distributed 1,400,000 syringes in DIC and outreach teams that provide harm reduction services over 6 months [50]. Therefore, this coinfection should decrease. Nevertheless, based on other countries' previous experiences, harm reduction programs for HCV transmission in IDU people are less effective than for those with HIV. This is probably due the higher transmission of HCV and the larger number of HCV-infected patients in the IDU population [12]. Timely diagnosis of HCV infection is also of great significance [12]. In the next stage, the effective treatment of these patients, as secondary prevention, is quite influential; however, it was not widely performed in the population under our investigation.

Surveys show that less than 10% of the 170 million HCV infected patients around the world are aware of their disease. As man is the only reservoir of HCV, elimination of the virus is not that far-fetched. Moreover, in contrast to HBV and HIV, there is no latent reservoir for this virus; and when a patient is cured, the danger of transmission of the virus to others is eliminated. If the environmental reservoir is sufficiently decreased, the infection cannot be transmitted easily, allowing the virus transmission cycle to be broken easily using proper disinfectants [12]. The present study had some limitations. Since HCV RNA could not be detected routinely in the population under study, HCV infection could only be diagnosed by serology, based on HCV Ab. Of course, this could not strongly affect the results of our study, since at least 1 year had passed from the participants' HIV infection and their entrance into the cohort. Further, the sensitivity of HCV Ab is over 99% [51]. On the other hand, as others have mentioned, serological study of HCV infections might underestimate the effect of HCV on AIDS-related complications. Therefore, had we measured HCV RNA, we would have observed a stronger relationship between HCV infection and AIDS-related deaths [51]. The second limitation was that the role of some infections, such as GB virus C and transfusion transmitted virus (TT Virus), was not investigated with regard to the relationship between HCV infection and AIDS-related deaths, since they were not routinely measure in our study population. As others have also shown, GB virus C leads to a decrease in HIV replication and improvements in HIV patients' survival. On the other hand, it can be sexually transmitted, and its prevalence is the same in infected people, IDU people, and other groups. Therefore, this virus cannot have altered the relationship between HCV infection and AIDS-related deaths [19].

Third, we did not know the exact time of HIV and HCV infection; thus, we considered the time of the laboratory exam as the time of infection. However, this moot point can change the actual impact of HCV on HIV/AIDS-related endpoints. Although our study revealed that HCV infection was diagnosed after HIV infection and entrance of the subjects into the study in nearly 90% of subjects, the exact time of HCV infection is not known. Fourth, because subjects entered our study at different stages of HIV/AIDS, it was very difficult to control for disease state on survival estimations. Fortunately, our evaluation revealed that because the center is unique in Shiraz, nearly all of our subjects were in the initial stages of infection. Besides, the impact of disease progression on survival was controlled by the baseline and trend of CD4 counts. The study sample was selected from a large population of HIV patients who solely and inevitably demand the country's health and treatment centers to treat their disease. These patients are from different cultural, social, and economic backgrounds. Further, because this center is the only provider of such services in Fars and some parts of southern Iran, this sample is a proper representative of the population of HIV patients in southern Iran. Moreover, regarding the results of other studies and considering the similarities between our study population and other parts of Iran, this sample can also be a proper representative of the entire country [52]. HAART treatment in the Iranian study population obviously improved HIV patients' survival. After controlling for the effect of HAART treatment, HCV infection resulted in an increase in AIDS-related deaths. In addition, our study enjoys a major practical application: it emphasizes the necessity of timely diagnosis and treatment, as well as serious harm reduction programs, in order to control and eliminate HCV infection and transmission.
